# High Incidence of Human Rabies Exposure in Northwestern Tigray, Ethiopia: A Four-Year Retrospective Study

**DOI:** 10.1371/journal.pntd.0005271

**Published:** 2017-01-06

**Authors:** Gebreyohans Gebru Teklu, Teweldemedhn Gebretinsae Hailu, Gebremedhin Romha Eshetu

**Affiliations:** 1 Department of Animal Sciences, College of Agriculture, Aksum University, Shire Campus, Shire, Ethiopia; 2 Department of Animal Production and Technology, College of Agriculture and Environmental Science, Adigrat University, Adigrat, Ethiopia; Wistar Institute, UNITED STATES

## Abstract

**Background:**

Rabies is a fatal zoonotic disease that has been known in Ethiopia for centuries in society as “Mad Dog Disease”. It is an important disease with veterinary and public health significance in the North western zone of Tigray where previous studies have not been conducted. Frequent occurrence of outbreaks in the area led the researchers to carry out a four year retrospective study to estimate the incidence of human rabies exposure in Northwestern Tigray, Ethiopia.

**Methodology:**

A referent study was conducted on human rabies exposure cases recorded from 2012 to 2015 at Suhul hospital, Shire Endaselase, Northwestern Tigray, Ethiopia. Exposure cases included in this research constituted victims bitten by unprovoked dogs and who received post exposure prophylaxis (PEP) at the hospital. Two thousand one hundred eighty human rabies exposure cases retrieved from the rabies case database were included in this study.

**Principal findings:**

The majority of the exposed cases were males (1363/2180, 63%). Age wise, the most exposed age group was ≥15 years in all the study years: 166 (58%), 335 (65%), 492 (66%) and 394 (63%) in 2012, 2013, 2014 and 2015, respectively. Similarly, exposure cases for human rabies increased with age in both males and females across the study years. The incidence of human rabies exposure cases calculated per 100,000 populations was 35.8, 63.0, 89.8 and 73.1 in 2012, 2013, 2014 and 2015, respectively. Binary logistic regression analysis revealed that being male was a risk for human rabies exposure in all the study years.

**Conclusion:**

The study discovered the highest annual human rabies exposure incidence in Ethiopia. This suggests an urgent need for synergistic efforts of human and animal health sectors to implement prevention and control strategies in this area.

## Author Summary

Rabies is a deadly disease of human and animals. The disease has been recognized in Ethiopia for centuries, but its impact remained underestimated. This limitation masks the true magnitude of rabies incidence and has been a stumbling block for its prevention and control. The aim of the study was to determine the incidence of human rabies exposure in Northwestern Tigray, Ethiopia, where studies have not been conducted, although outbreaks of the disease were common. A health facility-based four year referent study was conducted on human rabies exposure cases recorded from 2012 to 2015 at Suhul hospital, located in Shire Endaselase, Northwestern Zone of Tigray. Majority of the exposed cases were males. The most exposed age group was ≥15 years in all the study years. Similarly, exposure cases increased with age in both male and female individuals across the study years. Incidence of human rabies exposure cases per 100,000 populations also showed a continuous increment with the highest being recorded in 2014. Moreover, the study discovered the highest annual human rabies exposure incidence in Ethiopia. This suggests an urgent need for synergistic efforts of human and animal health sectors to implement prevention and control strategies.

## Introduction

Rabies is a fatal and one of the most important reemerging zoonotic diseases throughout the world, caused by RNA viruses that affect the central nervous system of all warm-blooded animals, including humans [1, 2]. Carnivores are one of the primary virus reservoirs and rabid dogs pose the greatest hazard of rabies worldwide [3]. Transmission of the virus usually occurs by the bite of rabid animals. Under unusual circumstances, inhalation of aerosolized virus and organ transplantation from rabid patients may occur [4]. Rabies is incurable once the clinical signs of the disease appear [5]. The virus penetrates the body through wounds or by direct contact with mucosal surfaces, replicates locally, gains centripetal access to the peripheral and central nervous system via motor endplates and motor axons [6] and undergoes centrifugal spread to major exit portals, such as the salivary glands [3] and excretion in saliva [2] which contains abundant virus and is the main source of infection.

Globally, rabies is estimated to cause more than 1.9 million disability-adjusted life years (DALYs) and 6 billion in annual monetary losses [2]. Although effective vaccines are widely available for humans and animals [7], rabies remains the most deadly neglected disease in developing countries [8, 9]. In Africa, an estimate of the rabies burden in humans is about 23,800 deaths and 609,000 DALYs [2]. It is also a cause of substantial livestock losses [10] and is a constant threat to rare carnivores such as the Ethiopian wolf (*Canis simensis*) [11] and the African wild dog (*Lycaon pictus*) [12]. In Ethiopia, rabies has been known for centuries in society as “Mad Dog Disease” [13], and has been recorded scientifically since 1903 [14]. To date, rabies is an important disease in Ethiopia both in human and animals [5, 15–19].

Conducting regular epidemiological surveillance programs, designing and enforcing laws for registration, certification and regular vaccination of owned dogs, creating public awareness as well as provision of easily accessible, effective and affordable post exposure human vaccines could result in efficient prevention and control of the disease [20]. In Ethiopia, however, lack of comprehensive national epidemiological data on rabies in animals and humans [21] results in underestimating the disease, which is a stumbling block for its control and prevention. This masks the true magnitude of disease incidence and reduces the efficiency of the notification system as well as surveillance potential [22] and hence reduces the concern of policy-makers and funding agencies. Historically, the incidence of human rabies exposure in Ethiopia ranges from 1.3 to 18.6 per 100,000 populations [13, 17, 21, 23].

However, most of these studies are 20 years old, which typically represent national data, confined in and around the capital city [5, 15]. Severe under-reporting of human rabies cases as well as lack of record keeping of the disease were reported in Eastern Ethiopia [19]. The latest national surveillance data of human rabies exposure cases (national incidence of 3.4/100,000), collected from all regional states, indicated that the highest incidence was registered in Addis Ababa (14.5/100,000), followed by Tigray (12.6/100,000) and Oromia (3.8/100,000) [21].

There is lack of scientific information on the status of human rabies and little is known about the disease to apply effective control measures in Northwestern Tigray. Frequent occurrence of outbreaks of human rabies and lack of documented information regarding the epidemiological situation of the disease led us to conduct this study. The objective of the research was to estimate the incidence of human rabies exposure in the Northwestern administrative zone, Tigray National Regional state, Ethiopia.

## Methods

### Study area description

**Fig 1:**
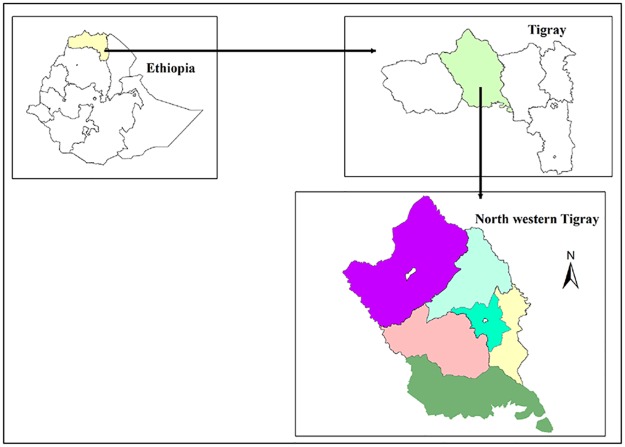
Map of the study area.

A health facility-based study was conducted from May to July, 2016 at Suhul hospital, located in Shire Endaselase, the capital city of the Northwestern Zone of Tigray (Fig 1). Shire Endaselase is located 1,087 km north of Addis Ababa, the capital city of Ethiopia. Suhul hospital is the only referral hospital that manages rabies cases and is the center for administration of post exposure prophylaxis (PEP) in Northwestern Tigray. The hospital accommodates patients from Shire and Sheraro towns as well as other six Woredas in the zone: Medebay Zana, Tahtay Koraro, Laelay Adiabo, Tahtay Adiabo, Tselemti and Asgede Tsimbla. The altitude ranges from 645 to 2852 meters above sea level. The total area is 18,325.1 km^2^. It receives an annual average rainfall of 877.6 mm, mainly from June to September, and temperature ranges from 18 to 34.6°C [24]. The main vegetation includes deciduous acacia species, *Eucalyptus* and riparian trees. Forest coverage of the area is estimated to be 40.5% (Zekarias, Agricultural advisor of Northwestern zonal administrator, personal communication, 2016). The Northwestern zone of Tigray is known for its livestock production potential. The Kafta Sheraro national park is in the study area, where 42 mammalian and 95 avian wildlife species reside. Elephants, spotted and striped hyenas, wild and serval cats, jackals, foxes, red fronted gazelle and greater kudu are common in the area.

### Study design and data collection

The study was conducted on human rabies exposure cases who registered from January 1, 2012 to December 31, 2015 for PEP. For convenience, we included only these four years because data of earlier years were not recorded appropriately and not easily accessible. Retrospectively, registered human rabies exposure cases of these four-year data were collected from the hospital’s annual disease report database. Moreover, discussion was held with the disease surveillance and control/prevention coordinator of Suhul hospital. Patients included in this study were kept anonymous to protect their medical confidentiality rights. Information retrieved comprised factors such as patient age and sex; species and vaccination history of rabies-suspect animals; history of exposure and previous rabies vaccination of the human cases; and recommended PEP for individuals. Two research team members retrieved the data from the hospital’s annual disease report database.

### Clinical diagnosis of suspected dogs

Prior to PEP administration for humans, a decision was made on suspect dogs which inflicted unprovoked bites through quarantine and follow up for 10 days, usually at the owner's premise and sometimes at the hospital compound. The dogs were diagnosed clinically as stated in the Standard Treatment Guidelines for General Hospitals [25] that unvaccinated dogs which cause unprovoked bites must be suspected to be rabid. The underlying principle is that rabies virus-infected animals could only transmit the virus shortly before and after clinical signs have developed. After quarantine, the dog might have either displayed a significant change in behavior or signs of illness suggestive of rabies and/or died during the observation period. Then, bite victims may have adequate time to receive PEP and prevent the disease development. By using the above principles, dogs suspected as rabid during the observation period have been positive for rabies virus by laboratory diagnosis [5]. ‘Suspected’ refers to a case that is compatible with a clinical case definition [3]. Unfortunately, confirmation of suspected dog brain samples by laboratory diagnosis was not practiced in the hospital due to a lack of facilities.

### Human rabies exposure and population at risk

After clinical diagnosis of the dog, the type of exposure was identified and human PEP was recommended. To identify the type of exposure, the definition of exposure used by the hospital was consistent with the European Centre for Disease Prevention and Control (ECDC) recommendation [26] and included both category II (minor exposure) and category III (severe exposure) exposures. Category II refers to minor scratches or abrasions without bleeding or licks on broken skin and nibbling of uncovered skin, while category III refers to single or multiple transdermal bites or scratches by infected animals. To collect history of human exposure, the hospital used a similar format developed by WHO [2] for cases of possible rabies exposure (S1 Text). A final decision for PEP was made based on history of exposure, typical clinical signs of the disease in dog and/or its death. When victims were bitten by stray dogs, it was difficult to follow up and the persons received PEP depending on the type of exposure. Exposure cases included victims bitten by unprovoked dogs and who received a complete PEP course at Suhul hospital. The human populations at risk (for the respective years, 2012–2015) of this study were estimated by projecting the results of the National Population and Housing Census of Ethiopia conducted in May 2007 [27].

### Ethical approval

Ethical approval was obtained from Aksum University, Research and Ethical Review Committee (S2 Text). Permission was sought from the hospital administration before data collection. Moreover, an official letter was issued to Suhul hospital that the findings would be used for scientific purposes.

### Statistical analysis

Data were entered, checked and analyzed using STATA statistical software (version 11.0,Stata Corp, college station, Texas 77845 USA). To ensure quality, data were entered and cross-checked independently by two members of the research team. Age, sex and time (year) based distribution of human rabies exposure cases was analyzed using descriptive statistics. With 95% confidence intervals, crude odds ratio was employed to compare the association between the outcome (human rabies exposure) and potential predictor variables (male and female) using binary logistic regression model. A P-value < 0.05 was considered statistically significant.

## Results

In total, 2180 human rabies exposure cases were registered and followed for their PEP at Suhul hospital from 2012 to 2015. The results showed that the number of human rabies exposure cases sharply increased in the study years with the highest being recorded in 2014. Dog bite was the only described cause of human rabies exposure in the study area.

### Demographic characteristics of human rabies exposure cases

Demographic data of the registered human rabies exposure cases (n = 2180) originated from two towns and six other administrative divisions (e.g., Woredas) of Northwestern Tigray were obtained and included in the study. Most of the exposed individuals were males (1363/2180, 62.5%). In all the four years, human rabies exposure cases were higher in males than females (Table 1). In the hospital rabies database, victim age was obtained clustered in three categories: ≤ 4, 5 to 14 and ≥15 years. Accordingly, the greatest exposed age group was ≥15 years in all the study years: 166 (58%), 335 (65%), 492 (66%) and 394 (63%) in 2012, 2013, 2014 and 2015, respectively. Similarly, it was observed that exposure for human rabies increased with age in both males and females across the four years (Table 1).

**Table 1:** Demographic distribution of human rabies exposure cases received PEP at Suhul hospital during 2012, 2013, 2014 and 2015.

Year	Age	Sex		Total
		Male	Female	
2012	< 4	22 (7.6%)	16 (5.6%)	38 (13.2%)
	5 to 14	56 (19.4%)	28 (9.7%)	84 (29.2%)
	> 15	98 (34.0%)	68 (23.6%)	166 (57.6%)
	Total	176 (61.1%)	112 (38.9%)	288 (100.0%)
2013	< 4	38 (7.4%)	24 (4.7%)	62 (12.0%)
	5 to 14	83 (16.1)	35 (6.8%)	118 (22.9%)
	> 15	206 (40.0%)	129 (25.0%)	335 (65.0%)
	Total	327 (163.5%)	188 (36.5%)	515 (100.0%)
2014	< 4	57 (7.6%)	36 (4.8%)	93 (12.4%)
	5 to 14	103 (13.8%)	59 (7.9%)	162 (21.7%)
	> 15	316 (42.3%)	176 (23.6%)	492 (65.9%)
	Total	476 (63.7%)	271 (36.3%)	747 (100.0%)
2015	< 4	34 (5.4%)	34 (5.4%)	68 (10.8%)
	5 to 14	106 (16.8%)	62 (9.8%)	168 (26.7%)
	> 15	244 (38.7%)	150 (23.8%)	394 (62.5%)
	Total	384 (61.0%)	246 (39.0%)	630 (100.0%)

### Incidence of human rabies exposure cases and its trend across the study years

**Fig 2:**
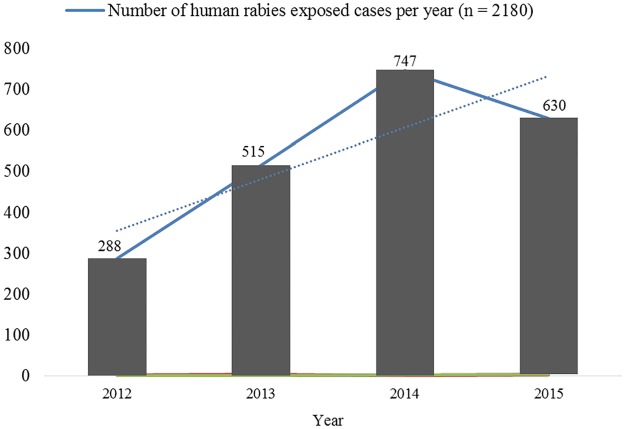
Trends of human rabies exposure cases across the study years.

The results showed that human rabies exposure cases increased across the study years with the highest recorded in 2014. The four-year trend of human rabies exposure cases is shown in Fig 2. The incidence of human rabies exposure cases calculated per 100,000 populations was 35.8, 63.0, 89.8 and 73.1 in 2012, 2013, 2014 and 2015, respectively. Binary logistic regression analysis revealed that being male was a risk for human rabies exposure in all study years (Table 2).

**Table 2:** Incidence of human rabies exposure cases registered and followed up their PEP at Suhul hospital in 2012, 2013, 2014 and 2015.

Year	Sex	Total Population	No. of exposure cases	Incidence per 100,000 population	COR (95% CI)	P-value
2012	Male	401,762	176	43.8	1.6(1.241–1.994)	<0.000
	Female	402,086	112	27.9		
	Total	803,848	288	35.8		
2013	Male	408,821	327	80	1.7(1.455–2.084)	<0.000
	Female	409,151	188	46		
	Total	817,972	515	63		
2014	Male	416,004	476	114.4	1.8(1.515–2.042)	<0.000
	Female	416,340	271	65.1		
	Total	832,344	747	89.8		
2015	Male	430,751	384	89.2	1.6(1.332–1.834)	<0.000
	Female	431,098	246	57.1		
	Total	861,849	630	73.1		

**COR**: Crude Odds Ratio

### Dog vaccination and human prophylaxis

Data on the coverage of preventive dog vaccination and demography were not evident in the study area. In this study, however, it was confirmed that all dogs inflicted unprovoked bites were unvaccinated. Moreover, inadequate supply of preventive dog vaccination was observed in the study area and was irregular in application. Victims who visited the nearest health centers were always sent to Suhul hospital and allowed to get the recommended doses of PEP. The PEP doses given, for category II and III exposures, were 14 doses for the first 14 days, consecutively, and then 3 doses at 10 days interval (i.e. 24^th^, 34^th^ and 44^th^ days). No rabies immune globulin (RIG) was administered. The attenuated Fermi vaccine produced by the Ethiopian Health and Nutrition Research Institute, Addis Ababa was used for PEP. Besides vaccine, standard wound management and administration of Tetanus antitoxin (TAT) was practiced. According to the manager of the hospital, Northwestern zone of Tigray was among the areas where rabies was the most prevalent. He also added that out of the total annually allocated PEP to Tigray National Regional State, nearly 60% was utilized by Suhul hospital.

## Discussion

Rabies is an entirely preventable infectious disease if regular epidemiological surveillance programs are practiced accompanied with enforcing laws for dog registration, vaccination and certification, and provision of easily accessible, effective and affordable vaccines and RIG [20]. It is an important disease with significant public health concern in the Northwestern zone of Tigray region. Human rabies exposure cases (n = 2180) registered for and followed up their PEP at Suhul hospital during the respective study years (2012, 2013, 2014 and 2015) showed a sharp increase with the highest recorded in 2014. This observation might be either from the improvement of the coverage of dog vaccination or under reporting conditions occurred in 2015. Binary logistic regression analysis revealed that being male was a risk for human rabies exposure in all the study years. This might be associated with activities of males, in that they are engaged in outdoor activities while females are more likely to remain indoors due to sociocultural and religious reasons. The same findings were reported in other locations of Ethiopia [5, 17] and Nigeria [28]. Age wise, most of the cases were found in people aged 15 years old and above (Table 1). The result was discordant with study conducted at Gondar, Ethiopia [17]. This might be explained by the wide range of clustering age. This suggests the extent of the impact of the disease on the national economy by affecting the most productive age group.

The incidence of human rabies exposure cases calculated per 100,000 populations was 35.8, 63.0, 89.8 and 73.1 in 2012, 2013, 2014 and 2015, respectively. This incidence was much higher than previously reported studies undertaken in Gondar areas, Ethiopia [16, 17]. So far, the incidence of human rabies exposure cases were commonly estimated from health facility records based on provision of PEP, which showed increases in the number of recorded human rabies exposure cases from 1986 onwards, ranging from 1.3 to 18.6 per 100,000 populations [13, 17, 21, 23]. The high human rabies exposure incidence of the present study reveals that the number of human rabies exposed individuals visited health facilities has increased. This might be due to growing awareness of the communities in the study area and/or increased risk of the disease resulted from ecological changes in the area.

However, recent studies conducted on rabies in different settings of Ethiopia showed that community awareness (knowledge) towards the disease was improved despite medical care seeking behavior was low. For instance, immediate follow up modern medication after bite and vaccination of dogs are not satisfactory. In addition, strong believe in traditional medicine for the treatment of rabies has been discovered. This indicates low level of community attitude towards the disease [16, 18, 29, 30]. In Eastern Ethiopia, one study confirmed that majority of the respondents have heard about the disease from their family in both urban and pastoralist households [19] which implied government based awareness creation might not be adequate or practiced. In the same study, overall poor knowledge about the disease has been reported in pastoralists. It has been demonstrated that investment in mass vaccination of owned dogs [8] and control of stray dogs [31]) are the most effective way of reducing the burden of rabies in humans. This is because canine rabies causes around 59,000 human deaths globally [8] and 95.6–96.6% of deaths from rabies occur in Asia and Africa, where canine rabies is enzootic [2]. In the present study, dog bite was the only source of rabies exposure. Similarly, 92% of humans who received post exposure anti-rabies treatments in Ethiopia were due to dog bites [15]. Likewise, other studies reported dog bite was the most important sources of human rabies [5, 16, 17, 29, 30].

In Africa, owner charged vaccination coverage is generally low though the percentage of dogs vaccinated under free of charge vaccination schemes is high [32]. According to the WHO recommendation [20], 70% of the dog population must be regularly vaccinated to control rabies. In Ethiopia, however, data on dog demography is not yet established [5]. Besides, the coverage of anti-rabies dog vaccination was not sufficient to put the disease under control [5, 21]. In the Awash Basin of Eastern Ethiopia, preventive dog vaccination was non-existent due to lack of availability of the vaccine [19]. The supply of preventive dog vaccination was also inadequate and irregular in the study area. This might signify that rabies, the tropical neglected zoonotic disease, has also not given attention in Ethiopia.

In the present study, only one fatal case was recorded at Suhul hospital in 2014 which seems to be insignificant. However, this was hospital based study and it is assumed that considerable numbers of the victims might have not visited health care posts because of the socio-cultural influences. Tschopp et al. [19] reported that individuals exposed to rabies could not visit health centers due to distance to health facility, mistrust in the medical system and poor knowledge about the disease. Thus, it is believed that the burden of rabies may be beyond the reported incidences because active surveillance has rarely been conducted. In Tanzania, it has been predicted that the incidence of human rabies, on the basis of active surveillance is 100 times greater than that of officially recorded [33]. In Ethiopia, severe under-reporting of human rabies cases as well as lack of record keeping of the disease were reported [19].

In Ethiopia, rehabilitation and biodiversity protection and management are among the strategies designed for poverty reduction [34, 35]. The study area (Northwestern Tigray) is known for its extensive uncultivated land with considerable forest coverage, which is estimated to be about 40.5% (Zekarias, agricultural advisor of Northwestern zonal administrator, personal communication, 2016). In Kafta Sheraro National Park wildlife might be reservoirs of the virus, as suggested in the Ethiopian Health and Nutrition Research Institute proceedings [21]. Due to wildlife concerns, most farmers/residents prefer to have more than one dog to guard their house. Opportunities for spillover infection at the wildlife-livestock/pet animal interface are a common phenomenon. This may facilitate circulation of viruses in the area. Wildlife are often believed to play a major role in transmission [2, 21]. In Ethiopia, one study identified that domestic dogs and cattle exhibiting clinical signs consistent with rabies, and people bitten by suspected rabid dogs, were reported in communities adjacent to Bale Mountains National Park, when rabies outbreak had occurred in wolves of this park [11]. Dog and livestock vaccination would reduce this concern for all species.

This study incorporated data of human rabies exposure case records inflicted by suspected dogs which had clinical information of their signs of illness suggestive of rabies. Nevertheless, sample submission for confirmation was not undertaken and the study was based on retrospective data. Nonexistence of laboratory confirmed cases in the hospital’s record was due to lack of facilities in the region. Moreover, the study did not include suspect or probable human rabies cases. Despite having these limitations, we believe that exposed humans (with exposure category II and III) had a high probability of developing rabies as all suspected dogs were not vaccinated. This is supported by research findings discovered at the Ethiopian Health and Nutrition Research Institute, in that dogs confirmed as suspect cases during the observation period have been found positive for rabies virus by laboratory findings [5]. In addition to the lack of rabies confirmation in suspect animals, no RIG was administered in situation of category III exposures, in conflict with current WHO recommendations for human rabies PEP [2].

In conclusion, the present study showed that human rabies exposure has shown a continuous increase in Northwestern Tigray across the study years (2012–2015) with the highest recorded in 2014. Moreover, the study discovered the highest annual human rabies exposure incidence in Ethiopia. This suggests an urgent need for synergistic efforts of human and animal health sectors to implement prevention and control strategies in this area.

## Supporting Information

S1 Text Data collection format. (PDF)

S2 Text Confirmation of ethical clearance. (PDF)

S3 Text Letter of support issued by Suhul hospital. (PDF)
