# Supplementary material for: High Incidence of Human Rabies Exposure in Northwestern Tigray, Ethiopia: A Four-Year Retrospective Study
Source: PLoS Negl Trop Dis. 2017 Jan 6;11(1):e0005271. doi: 10.1371/journal.pntd.0005271 (PMC5245898; doi:10.1371/journal.pntd.0005271)
Supplement: S1 Text — (PDF) [file pntd.0005271.s001.pdf]

Rabies data collection format

I. Human rabies exposure cases

1. Case no.:_______ Date: __________ Time: __________

2. Name: _________________
3. Age: a) ≤ 4 b) 5 to 14 and c) > 15
4. Sex: a) male b) Female
5. Address: a) district____________ b) Kebelle______________ Telephone:______________
6. Details of exposure
   - a) Place of exposure:___________________
   - b) Date of exposure:___________________
   - c) Nature of exposure: a) bite b) lick c) saliva d) scratch e) other (specify)________________
   - d) Site of exposure:______________
   - e) Was the skin broken? a) Yes b) No
   - f) Did the wound/s bleed? a) Yes b) No
5. Number of wounds: _____________
6. Depth of bite/s: a) superficial b) deep
7. Category of exposure: a) I b) II c) III
8. Previous rabies vaccination history of the victim
   - a) Did he/she have pre-exposure rabies vaccination? a) Yes b) No
   - b) If yes, site of administration____________
   - c) Was anti-rabies post-exposure prophylaxis given previously? a) Yes b) No
   - d) Which rabies vaccine was given? ________Details (day/date etc.) _________
   - f) Site of vaccination: ________________
9. Recommended treatment
   - a) Wound washing using water/ soap/ antiviral agent.
   - b) Vaccination for those with previous pre-exposure prophylaxis:____________
   - c) Standard course for unvaccinated:_____________________
10. Patient weight (kg): __________________

II. Details of the biting animal

1. Type of animal: a) Wild b) domestic
2. Species of animal: _______________
3. Was the animal provoked/unprovoked? Give details:_____________________
4. Is the animal's owner/home known? a) Yes b) No
5. If the answer for no.4 is No, what were the efforts made to trace the animal? Give details_______
6. Animal's previous vaccination status, if known _____________________________
7. What was previous and current (at the time of bite) behavior of the animal if known? _________
8. What was the condition of the animal during the observation a) Healthy and alive b) Displayed typical rabies clinical signs and euthanized c) Died after displayed typical rabies clinical signs
