# Supplementary material for: High Incidence of Human Rabies Exposure in Northwestern Tigray, Ethiopia: A Four-Year Retrospective Study
Source: PLoS Negl Trop Dis. 2017 Jan 6;11(1):e0005271. doi: 10.1371/journal.pntd.0005271 (PMC5245898; doi:10.1371/journal.pntd.0005271)
Supplement: S2 Text — (PDF) [file pntd.0005271.s002.pdf]

አክሱም ዩኒቨርሲቲ ሽረ ካምፓስ
ግብርና ኮሌጅ
ምርምርና ማ/ሰብ አ/ጉዳዮች ፅ/ቤት
✉ 314

Aksum University Shire Campus
College of agriculture
Research & community service affairs

ሽረ፣ ኢትዮጵያ **Shire, Ethiopia**

Date: 02 May 2016

To Whom It May Concern

Ref: **Confirmation of Ethical Clearance**

The Research and Ethics Review Committee (RERC) of Aksum University Shire Campus has critically evaluated the scientific and ethical aspects of the research proposal titled **"Incidence of Human Rabies Exposure in North western Zone of Tigray, Ethiopia"** to be conducted by Dr. Gebreyohans Gebru and Teweldemedhn Gebretinsae at Suhul Hospital of Shire Endaselase. Thus the committee has approved the proposal on the following conditions:

1. An official letter must be issued to Suhul Hospital to aware that the findings would be used for scientific purposes and must agree to provide the research data,
2. The comments forwarded are fully addressed,
3. The hospital must provide letter of confirmation about the data given to the researchers and
4. The research and community service officer makes sure that the research is conducted according to the approved proposal.

With Best Regards,

Kiros Gebretsadik
Research and community service officer and Chairman of RERC
