# Supplementary material for: High Incidence of Human Rabies Exposure in Northwestern Tigray, Ethiopia: A Four-Year Retrospective Study
Source: PLoS Negl Trop Dis. 2017 Jan 6;11(1):e0005271. doi: 10.1371/journal.pntd.0005271 (PMC5245898; doi:10.1371/journal.pntd.0005271)
Supplement: S3 Text — (PDF) [file pntd.0005271.s003.pdf]

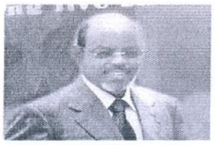

ሽረ ሆስፒታል ሱሑል

SUHUL HOSPITAL

SHIRE, Tigray, Ethiopia

ኣብ ቢሮ ሓላፊ ጥዕና ክልል [illegible]

ዞባ ሰሜን ምዕራብ

ሱሑል ሆስፒታል

ቁጥሪ 1773/[illegible]/08

Date 09 Aug 2016

ሕድርኽ ኣይንዕብርን

To whom it may concern

Ref: Confirmation letter on publication of data about rabies

As per their request with the attached data collection format, Suhul Hospital has given four years data, 2012 to 2015, on rabies to researchers of Aksum University. Our data is recorded in a digital database at the hospital. With this letter, I confirm the integrity of the data and our informed consent that the data can be used for publication in a scientific journal.

With kind regards,

Tesfazgi Gebrehiwot
Manager

☏ 03-44-44-25-61 M. Director
03-44-44-25-60 CEO
03-44-44-25-64 Personnel

E-mail Suhulhosp@yahoo.com

✉ 117 Shire Suhul Hospital, Tigray, Ethiopia

S.A
